# FKBP3 aggravates the malignant phenotype of diffuse large B‐cell lymphoma by PARK7‐mediated activation of Wnt/β‐catenin signalling

**DOI:** 10.1111/jcmm.18041

**Published:** 2023-11-21

**Authors:** Xiaojing Xing, Meichen Liu, Xuguang Wang, Qianxue Guo, Hongyue Wang, Wenxue Wang

**Affiliations:** ^1^ Department of Hematology and Breast Cancer Cancer Hospital of Dalian University of Technology (Liaoning Cancer Hospital & Institute) Shenyang China; ^2^ Department of Pathology Shenyang Medical College Shenyang China; ^3^ Department of Scientific Research and Academic Cancer Hospital of Dalian University of Technology (Liaoning Cancer Hospital & Institute) Shenyang China; ^4^ State Key Laboratory of Robotics, Shenyang Institute of Automation Chinese Academy of Sciences Shenyang China; ^5^ Institutes for Robotics and Intelligent Manufacturing Chinese Academy of Sciences Shenyang China

**Keywords:** DLBCL, FKBP3, FOXO3, lymphoma, PARK7, wnt/β‐catenin

## Abstract

Diffuse large B‐cell lymphoma (DLBCL) is difficult to treat due to the high recurrence rate and therapy intolerance, so finding potential therapeutic targets for DLBCL is critical. FK506‐binding protein 3 (FKBP3) contributes to the progression of various cancers and is highly expressed in DLBCL, but the role of FKBP3 in DLBCL and its mechanism are not clear. Our study demonstrated that FKBP3 aggravated the proliferation and stemness of DLBCL cells, and tumour growth in a xenograft mouse model. The interaction between FKBP3 and parkinsonism associated deglycase (PARK7) in DB cells was found using co‐immunoprecipitation assay. Knockdown of FKBP3 enhanced the degradation of PARK7 through increasing its ubiquitination modification. Forkhead Box O3 (FOXO3) belongs to the forkhead family of transcription factors and inhibits DLBCL, but the underlying mechanism has not been reported. We found that FOXO3 bound the promoter of FKBP3 and then suppressed its transcription, eventually weakening DLBCL. Mechanically, FKBP3 activated Wnt/β‐catenin signalling pathway mediated by PARK7. Together, FKBP3 increased PARK7 and then facilitated the malignant phenotype of DLBCL through activating Wnt/β‐catenin pathway. These results indicated that FKBP3 might be a potential therapeutic target for the treatment of DLBCL.

## 1 INTRODUCTION

Diffuse large B‐cell lymphoma (DLBCL), as a lymphoid malignancy, is the most common kind of non‐Hodgkin lymphoma (NHL) worldwide and characterized by aggressive and highly heterogeneous.^1^ Inheritance, viruses, several medical drugs and chemical substances, including alkylating agents, fertilizers and pesticides, have been considered to be the cause of DLBCL.^2^ Clinically, most patients usually exhibit rapidly growing lymphadenopathy and extranodal disease that require immediate treatment. However, patients with DLBCL relapse easily and are intolerable to medicine therapy.^3^ Approximately 30%–40% of patients who accepted the current therapies are still not cured.^4^ Therefore, it is important to find the potential therapeutic targets of DLBCL.

FK506‐binding proteins (FKBPs), as an immunophilin family, participate in the occurrence and progression of cancers.^5^ FKBP3 is a member of the FKBPs family and involves in the development of colorectal cancer (CRC) and non‐small cell lung cancer (NSCLC).^6^, ^7^ High expression of FKBP3 in DLBCL is observed in Gene Expression Profiling Interactive Analysis (GEPIA) database. Similarly, Uranishi et al. found that FKBP3 level was changed in B‐cell lymphomas.^8^ These findings suggest that FKBP3 may be associated with the development of malignant behaviour in DLBCL cells. As a basic member of Forkhead Box O (FOXO) family, FOXO3 regulated the proliferation of B lymphocytes and played role in patients with DLBCL by inhibiting tumour growth.^9^, ^10^ Juhász et al.^11^ proposed that in a FOXO3 overexpression background, the member of FKBPs family was downregulated. It revealed that the expression of FKBP3 would be inhibited by FOXO3.

In addition, parkinsonism associated deglycase (PARK7) is high expression in DLBCL which is analysed using GEPIA database. PARK7 involved in stemness of glioblastoma stem cells (GSCs), and origin and development of uveal melanoma, suggesting its importance in cancer.^12^, ^13^ Mechanically, the Wnt/β‐catenin signalling pathway was activated by PARK7, thereby promoting CRC progression and oesophageal squamous cell carcinoma proliferation, invasion and metastasis.^14^, ^15^ Activated Wnt/β‐catenin signalling pathway has been verified to exacerbate the malignant behaviour of DLBCL.^16^, ^17^, ^18^ After querying HitPredict, we found that FKBP3 may combine with PARK7. Besides, FKBP3 regulates the stability of the protein to which it binds by affecting the ubiquitination of the protein.^7^, ^19^ Therefore, we speculated that FKBP3 might influence PARK7 through regulating ubiquitylation of PARK7.

Here, the role of FKBP3 on malignant phenotype of DLBCL cells and its mechanism mediated by PARK7 were detected. The inhibitory effect of FOXO3 on the transcription of FKBP3 was verified.

## 2 MATERIALS AND METHODS

### 2.1 Cell culture and cell transfection

Human embryonic kidney (HEK)‐293 cells were purchased from iCell Bioscience Inc. (Shanghai, China) and cultured in minimum essential medium (Solarbio, Beijing, China) with 10% fetal bovine serum (FBS) at 37°C in 5% CO_2_ cell incubator.

DLBCL cell line DB and farage were provided by Procell (Wuhan, China) and cultured in RPMI Medium 1640 (Solarbio) with 10% FBS (Zhejiang Tianhang Biotechnology Co., Ltd., Huzhou, China). The cells were seeded in 6‐well plates and cultured for 24 h.

The cell lines have recently been authenticated by Short Tandem Repeat (STR).

For cell transfection, the cells were transfected with FKBP3‐flag overexpression vector (oeFKBP3) or negative expression vector (vector 1), PARK7‐HA overexpression vector (oePARK7) or negative expression vector (vector 2), FOXO3 overexpression vector (oeFOXO3) or negative expression vector (vector 4), FKBP3 siRNA (siFKBP3‐1, siFKBP3‐2, siFKBP3‐3 and siFKBP3‐4), PARK7 siRNA or negative control (siNC) using transfection reagent Lipo8000 (Beyotime Biotech Co., Ltd., Shanghai, China). The sequence of siFKBP3‐1 was sense: 5′‐GAAGCUUAAUGAAGAUAAATT‐3′, antisense: 5′‐UUUAUCUUCAUUAAGCUUCTT‐3′; siFKBP3‐2 was sense: 5′‐GAAGAUAAACCCAAAGAAATT‐3′, antisense: 5′‐UUUCUUUGGGUUUAUCUUCTT‐3′; siFKBP3‐3 was sense: 5′‐CAGAAUGGGCUUACGGAAATT‐3′, antisense: 5′‐UUUCCGUAAGCCCAUUCUGTT‐3′; siFKBP3‐4 was sense: 5′‐CAAACAAGUGCAAAGAAGATT‐3′, antisense: 5′‐UCUUCUUUGCACUUGUUUGTT‐3′; PARK7 siRNA was sense: 5′‐UGAAAUAGGUUUUGGAAGUTT‐3′, antisense: 5′‐ACUUCCAAAACCUAUUUCATT‐3′; siNC was sense: 5′‐UUCUCCGAACGUGUCACGUTT‐3′, antisense: 5′‐ACGUGACACGUUCGGAGAATT‐3′. After transfection with oeFKBP3 or shFKBP3 (5′‐GGAAGCTTAATGAAGATAAATTCAAGAGATTTATCTTCATTAAGCTTCTTTTT‐3′), and the stably transfected cells were selected by G418 (200 μg/mL; Solarbio).

### 2.2 Cell counting kit‐8 (CCK‐8) assay

At 0, 24, 48, 72 h after transfection, cell proliferation and viability were detected by a CCK‐8 kit (Biosharp, Hefei, China). The optical density (OD) value was measured by a microplate reader 800TS (BioTek, Winooski, VT, USA) at 450 nm.

### 2.3 5‐ethynyl‐2′‐deoxyuridine (EdU) assay

Forty‐eight hours after transfection, a EdU kit (KeyGEN, Nanjing, China) was used to analyse the cell proliferation. After redyeing with 4′,6‐diamidino‐2‐phenylindole (DAPI), the image was observed under a fluorescence microscope IX53 (OLYMPUS, Tokyo, Japan).

### 2.4 Cell cycle analysis

Forty‐eight hours after transfection, cells were fixed in pre‐cold 70% ethanol at 4°C overnight. Cell samples were analysed using a cell cycle detection kit (KeyGEN) under a flow cytometer NovoCyte (Agilent Technologies, Santa Clara, CA, USA).

### 2.5 Immunofluorescence

After preparation of cell slides, they were fixed in 4% paraformaldehyde for 15 min and then permeabilized with 0.1% tritonX‐100 (Beyotime Biotech Co., Ltd.) for 30 min at room temperature. After being blocked with 1% bovine serum albumin (BSA; Sangon Biotech, Shanghai, China), the cells were incubated with primary antibodies overnight at 4°C followed by the second antibodies for 60 min. DAPI (Aladdin) was used to stain cell nucleus. After adding anti‐fluorescence quenching agent (Solarbio), the immunofluorescent images were observed by a fluorescence microscope BX53 (OLYMPUS). Primary antibodies included prominin 1 (CD133) antibody (1:200; proteintech, Rosemont, IL, USA), FKBP3 antibody (1:100; Affinity, Changzhou, China) and PARK7 antibody (1:50; Santa Cruz, Dallas, TX, USA). Second antibodies included Cy3‐labelled goat anti‐rabbit IgG secondary antibody (1:200; Thermo Scientific, Pittsburgh, PA, USA) and FITC‐conjugated goat anti‐mouse IgG secondary antibody (1:200; Abcam, Cambridge, UK).

### 2.6 Co‐immunoprecipitation (CoIP)

Immunoprecipitation assay (RIPA) lysis buffer (Solarbio) with phenylmethylsulfonyl fluoride (PMSF; Solarbio) was used to lyse cells. Bicinchoninic acid (BCA) protein assay kit (Solarbio) was used to quantify the protein. AminoLink Plus Coupling Resin was incubated with Flag tag antibody (10 μg; proteintech) or HA tag antibody (10 μg; proteintech) for 120 min at room temperature on a rotator. The complex was incubated with the cell lysate for 2 h. After elution, the sample was loaded into sodium dodecyl sulfate‐polyacrylamide gel electrophoresis (SDS‐PAGE) for separation. The protein in the gel was transferred to polyvinylidene difluoride (PVDF) membrane (Millipore, Billerica, MA, USA). After being blocked with skimmed milk for 1 h at room temperature, membrane was incubated with primary antibodies overnight at 4°C and then incubated with secondary antibodies for 1 h at 37°C. Enhanced chemiluminescence (ECL) reagent was used to visualize the immunoblot bands, and optical density of bands was measured using Gel‐Pro‐Analyzer software. Primary antibodies included Flag tag antibody (1:5000; proteintech), HA tag antibody (1:5000; proteintech) and Myc tag antibody (1:2000; proteintech), and secondary antibodies included horseradish peroxidase (HRP)‐labelled goat anti‐rabbit immunoglobulin G (IgG) (1:3000; Solarbio) and HRP‐labelled goat anti‐mouse IgG (1:3000; Solarbio).

### 2.7 Luciferase reporter assay

When the cells grew to 90% confluency, HEK‐293 cells and DB cells were seeded in 12‐well plates. HEK‐293 cells were transfected with plasmids and Lipo8000 transfection reagent. Plasmids included TOPflash reporter (Addgene, Cambridge, MA, USA), pRL‐TK, vector 1 or oeFKBP3. In another experiment, the promoter region of FKBP3 was cloned into the pGL3‐enhancer reporter vector and then co‐transfected into DB cells with FOXO3 overexpression plasmid and pRL‐TK. After 48 h, cells were lysed for the luciferase assay and the assay was performed using a dual luciferase reporter gene assay kit (KeyGEN).

### 2.8 Chromatin immunoprecipitation (ChIP) assay

ChIP Kit (Wanleibio, Shenyang, China) was used to proceed the ChIP assay, following the manufacturer's instructions. For each ChIP, 2 μg of FOXO3 antibody (nouvs, Saint Charles, Missouri, USA), 1 μg of RNA PolymeraseII and 1 μg of normal IgG were used. IgG antibody was regarded as a negative control. After immunoprecipitation, DNA was purified and the FKBP3 promoter region was detected by using polymerase chain reaction (PCR). The reaction parameters were 95°C (for 10 s), 55°C (for 20 s) and 72°C (for 30 s) for 38 cycles. PCR product was electrophoresed in a 2.0% agarose gel and photographed with a gel imaging system WD‐9413B (BEIJING LIUYI BIOTECHNOLOGY Co., Ltd., Beijing, China). The primers used were forward: 5′‐GCTAACTCATAGGCAACA‐3′ and reverse: 5′‐CTCAGTACACGAGGAAAC‐3′.

### 2.9 Nude mouse xenograft model

All animal experiments were performed following the Guideline for the Care and Use of Laboratory Animals and approved by the Experimental Animal Ethics Committee of Shenyang Medical College. BALB/c nude mice (4–6 weeks, sex in half) were adaptively fed for 1 week. The nude mice were randomly divided into four groups (vector 1, oeFKBP3, shNC and shFKBP3). DB cells stably transfected with vector 1, oeFKBP3, shNC and shFKBP3 were mixed with an equal volume of matrigel and then injected subcutaneously into nude mice. One week after injection, tumour volume was measured every 4 days. Twenty‐one days later, mice were euthanized and tumour were obtained, weighed and photographed. Part of the tumours were frozen in liquid nitrogen and transferred to an ultra‐low temperature refrigerator at −70°C for storage, and part were fixed with 4% paraformaldehyde for subsequent experiments.

### 2.10 Immunohistochemistry (IHC)

Tumour was obtained from mice, fixed in 4% paraformaldehyde, dehydrated in ethanol, permeated with xylene, embedded in paraffin and then cut into 5‐μm‐thick section. Section was put into antigen repair solution, heated for 10 min and cooled to room temperature. To eliminate the endogenous peroxidase, the section was incubated with 3% H_2_O_2_ for 15 min. After being blocked with 3% BSA, the section was incubated with proliferating cell nuclear antigen (PCNA) antibody (Affinity) overnight at 4°C and then with HRP‐labelled goat anti‐rabbit IgG secondary antibody (Thermo Scientific) for 60 min at 37°C. After colouration with 3,3′‐diaminobenzidine (DAB; MXB Biotechnologies, Fuzhou, China) and redyeing with haematoxylin (Solarbio), successively, section was observed under a microscope BX53 (OLYMPUS).

### 2.11 Real‐time PCR

The total RNA was extracted using TRIpure (BioTeke Corporation, Beijing, China) and its concentration was measured by an ultraviolet spectrophotometer NANO 2000 (Thermo Scientific). BeyoRT™ II M‐MLV reverse transcriptase (Beyotime Biotech Co., Ltd.) and RNase inhibitor (Sangon Biotech) were used to synthesize cDNA. Gene expression was detected by a fluorescent quantitative PCR instrument Exicycler 96 (Bioneer Corporation, Daejeon, Korea) using SYBR Green (Solarbio). The mRNA expression was normalized to GAPDH and calculated using the $2^{-\Delta \Delta C_{t}}$ method. Primer sequences were described as follows: FKBP3 forward, 5′‐GATAAACCCAAAGAAACC‐3′ and reverse, 5′‐TACCAGCAGTGAACAACAT‐3′; Nestin forward, 5′‐ACCCTTGCCTGCTACCCT‐3′ and reverse, 5′‐AGCCTGTTTCCTCCCACC‐3′; CD133 forward, 5′‐CCAAGGACAAGGCGTTCA‐3′ and reverse, 5′‐GCACCAAGCACAGAGGG‐3′; POU class 5 homeobox 1 (OCT4) forward, 5′‐AGGGCAAGCGATCAAGC‐3′ and reverse, 5′‐GGAAAGGGACCGAGGAGTA‐3′; SRY‐Box transcription factor 2 (SOX2) forward, 5′‐ATGCACCGCTACGACGTGAG‐3′ and reverse, 5′‐GCCCTGGAGTGGGAGGAAGA‐3′; cycin D1 forward, 5′‐GCGAGGAACAGAAGTGCG‐3′ and reverse, 5′‐GGAGTTGTCGGTGTAGATGC‐3′; MYC Proto‐Oncogene, BHLH Transcription Factor (MYC) forward, 5′‐ACACCCTTCTCCCTTCG‐3′ and reverse, 5′‐CCGCTCCACATACAGTCC‐3′; PCNA forward, 5′‐CAAGAAGGTGTTGGAGGCA‐3′ and reverse, 5′‐TCGCAGCGGTAGGTGTC‐3′; FOXO3 forward, 5′‐TGACGACAGTCCCTCCC‐3′ and reverse, 5′‐GCTGGCGTTAGAATTGGT‐3′; GAPDH forward, 5′‐GACCTGACCTGCCGTCTAG‐3′ and reverse, 5′‐AGGAGTGGGTGTCGCTGT‐3′.

### 2.12 Western blot analysis

Protein was extracted by utilizing RIPA lysis buffer (Solarbio) with PMSF (Solarbio) and protein concentration was quantified using the BCA protein assay kit (Solarbio). Equal amount of protein was loaded into SDS‐PAGE and then separated. The protein in the gel was transferred to PVDF membrane (Millipore). After being blocked with skimmed milk for 1 h at room temperature, the membrane was incubated with primary antibodies overnight at 4°C and then with secondary antibodies for 1 h at 37°C. ECL reagent was used to visualize the immunoblotting bands, and the optical density of bands was measured by Gel‐Pro‐Analyzer software. Primary antibodies included FLAG (1:5000; proteintech), FKBP3 (1:1000; proteintech), PCNA (1:1000; proteintech), cyclin B1 (1:1000; Affinity), cyclin D1 (1:1000; Affinity), cyclin dependent kinase 4 (CDK4, 1:500; Affinity), CDK1 (1:500; Affinity), PARK7 (1:1000; proteintech), HA(1:5000; proteintech), active β‐catenin (1:1000; CST, Danvers, MA, USA), FOXO3 (1:500; nouvs, Saint Charles, Missouri, USA), GAPDH (1:10000; Affinity) and secondary antibodies included HRP‐labelled goat anti‐rabbit IgG (1:3000; Solarbio), HRP‐labelled goat anti‐mouse IgG (1:3000; Solarbio).

For protein degradation experiment, after transfection for 48 h, the cells were treated with cycloheximide (CHX, 100 μg/mL; MedChemExpres, Shanghai, China) with/without MG132 (10 μM; Aladdin, Shanghai, China) for 0, 1, 3, 6 and 9 h. PARK7 protein level was then determined using western blot analysis as described above.

### 2.13 Database

The dataset of Gene Expression Profiling Interactive Analysis (GEPIA) (http://gepia2.cancer‐pku.cn/#analysis) was used to analyse FKBP3 and PARK7 expression in DLBCL and normal samples. To predict possible functions and to define the pathways used by differently expressed genes (DEGs), GO and KEGG pathway analyses were performed.

### 2.14 Statistical analysis

Data were expressed as the mean ± standard deviation (SD). Differences among groups were analysed using t tests or One‐way anova. The analysis was performed with GraphPad Prism 8.0.2 and *p*‐value <0.05 was viewed statistically significant.

## 3 RESULTS

### 3.1 Bioinformatics analysis of DLBCL dataset in GEPIA

To explore potential gene targets for DLBCL and identify DEGs related to DLBCL, the DLBCL dataset in GEPIA was analysed. DEGs were identified as log_2_FC >2 or < −2 and *p* < 0.01. The heat map showed that the expression of DEGs in which FKBP3 and PARK7 were highly expressed in the tumour (Figure S1A). GO pathway analysis displayed that the DEGs were enriched in cytoplasmic translation, mitochondrial gene expression, rRNA processing, ribosome biogenesis, rRNA metabolic process and ribonucleoprotein complex biogenesis during biological process; in organellar ribosome, mitochondrial ribosome, ribosomal subunit, small ribosomal subunit, large ribosomal subunit and cytosolic ribosome in cellular component; in structural constituent of ribosome, rRNA binding, translation initiation factor activity, oxidoreduction‐driven active transmembrane transporter activity, translation factor activity, RNA binding and translation regulator activity, nucleic acid binding in molecular function (Figure S1B). DEGs were mainly related to the DNA replication, ribosome, proteasome, base excision repair, cell cycle and nucleocytoplasmic transport in KEGG analysis (Figure S1C). The exact location of DEGs, including FKBP3, on chromosomes was shown in Figure S1D. All data indicated that FKBP3 was highly expressed in DLBCL.

### 3.2 FKBP3 promoted the proliferation of DLBCL cells

**FIGURE 1:**
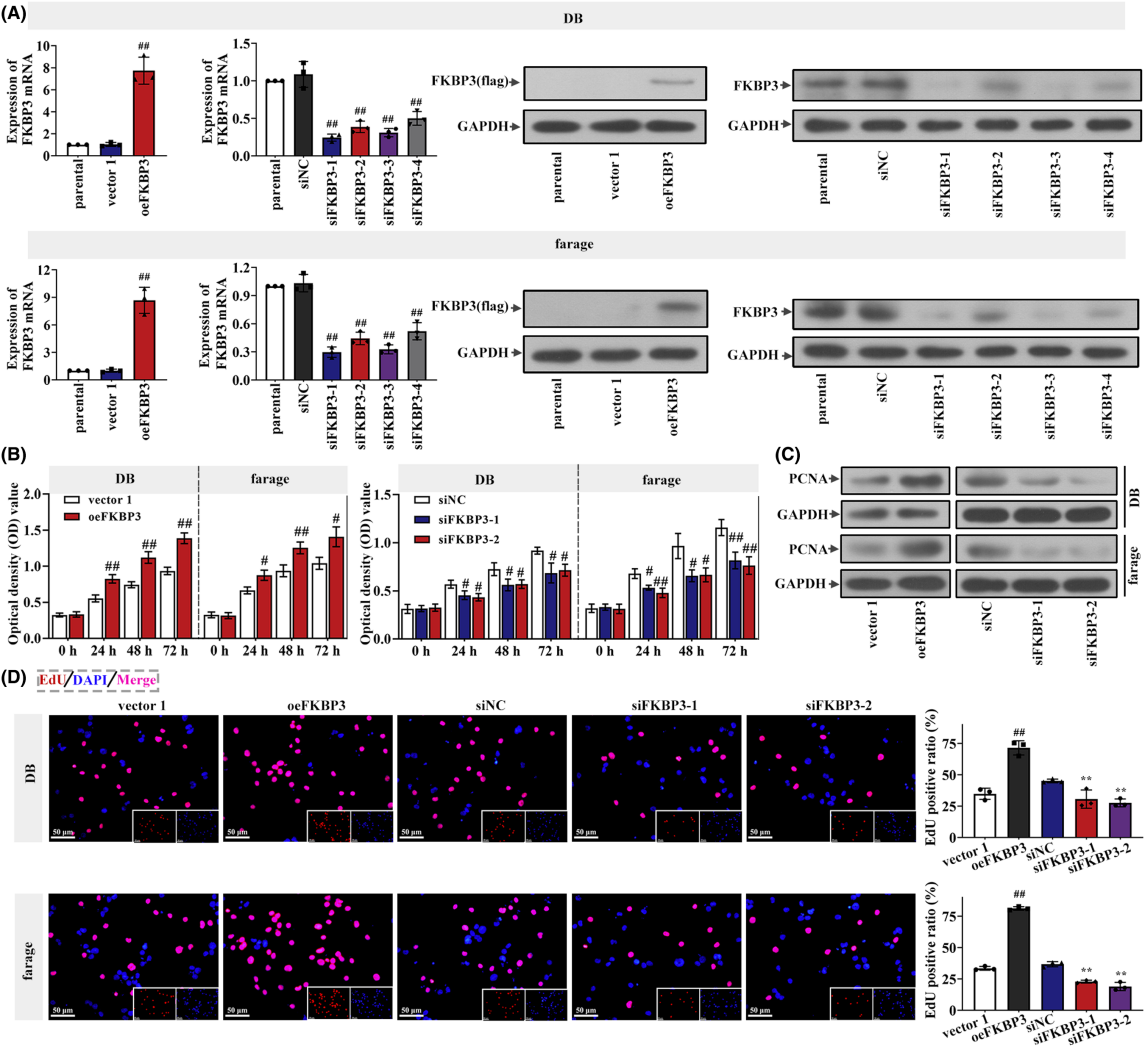
FKBP3 promoted the proliferation of DLBCL cells. (A) Real‐time PCR and western blot analysis were used to detect the mRNA and protein expression of FKBP3 in DB cells and farage cells. (B) Cell viability analysis was performed by CCK‐8 after transfection 0, 24, 48 and 72 h. The optical density value meant the cell viability. (C) The protein expression of PCNA in DB cells and farage cells was detected by western blot analysis. (D) EdU in DB cells and farage cells measured by a fluorescence microscope IX53. EdU‐incorporated signal was red and nucleus was stained blue with DAPI in DB cells and farage cells. Bars, 50 μm, ×400. Data were expressed as mean ± SD (*n* = 3). ^#^ *p* < 0.05 compared to vector 1 or siNC. ^##^ *p* < 0.01 compared to vector 1 or siNC. **P < 0.01 compared to siNC. CCK‐8, cell counting kit‐8; DAPI, 4′,6‐diamidino‐2‐phenylindole; DLBCL, diffuse large B‐cell lymphoma; EdU, 5‐ethynyl‐2′‐deoxyuridine; FKBP3, FK506 binding protein 3; PCNA, proliferating cell nuclear antigen; PCR, polymerase chain reaction; SD, standard deviation.

Based on the data from GEPIA, we speculated that FKBP3 might be an oncogene in DLBCL and determined its function through experiments. After successfully overexpressing and silencing FKBP3 (Figure 1A), we investigated whether FKBP3 regulated the growth of DLBCL cell and for the first time detected the proliferation changes of DLBCL cells infected by oeFKBP3 or siFKBP3. After 24, 48 and 72 h of transfection, the OD value was significantly increased in oeFKBP3 transfected cells but decreased markedly in cell transfected with siFKBP3 (Figure 1B). These data suggested that FKBP3 enhanced cell viability, but on the contrary, FKBP3 silencing impaired cell viability. PCNA protein expression, a marker of cell proliferation, was prominent in oeFKBP3 transfected cells, but less so in siFKBP3 transfected cells (Figure 1C). Cells transfected with oeFKBP3 showed more EdU‐incorporated signals. In siFKBP3 transfected cells, EdU‐positive cells were observably decreased (Figure 1D). These results revealed that FKBP3 was beneficial for the growth of DLBCL cells.

**FIGURE 2:**
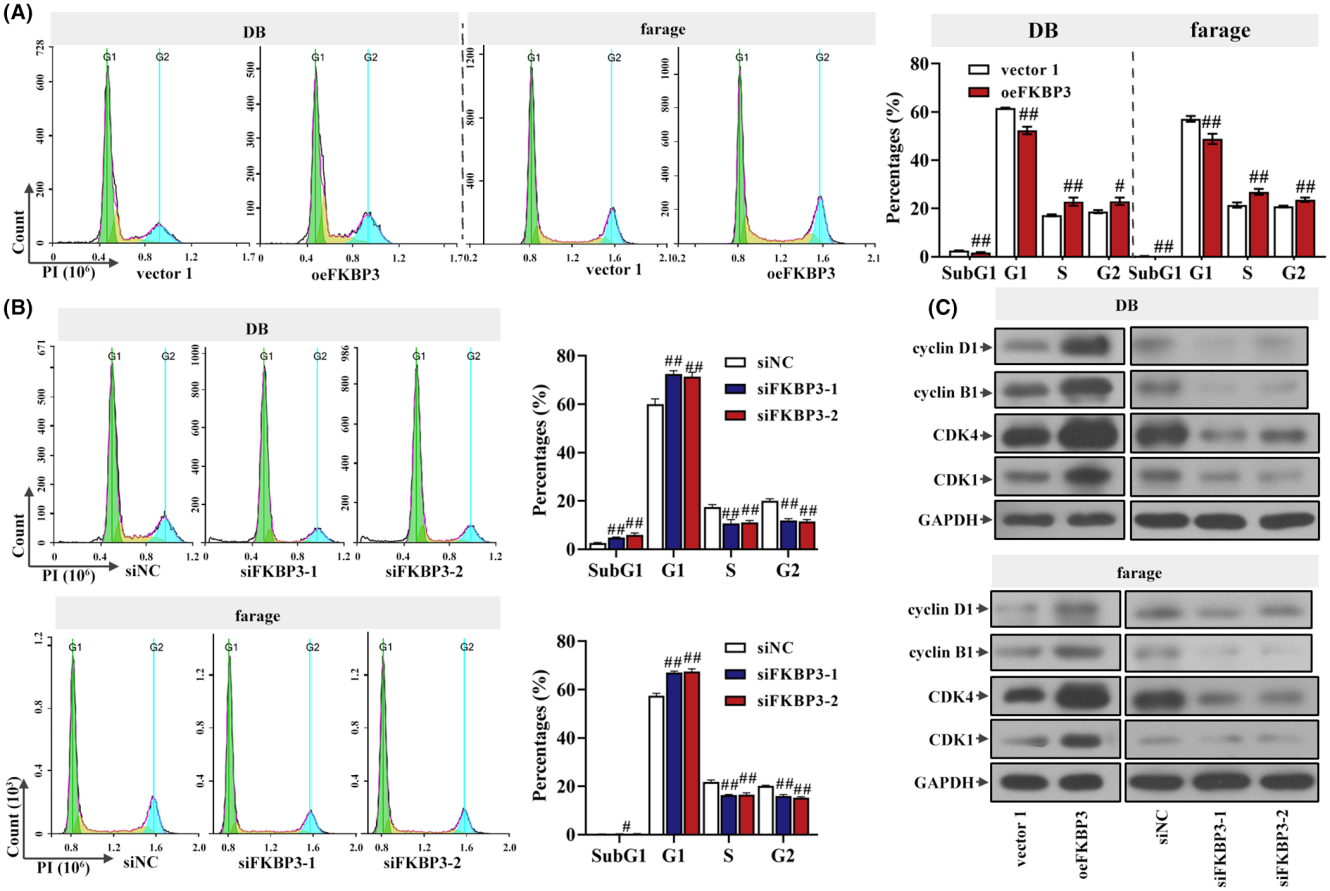
Knockdown of FKBP3 induced G1 arrest of DLBCL cells. (A) The effect of FKBP3 overexpression on cell cycle was detected by a flow cytometer NovoCyte. The PI was used to dye. (B) Cell cycle was analysed using flow cytometry in DB and farage cells with silenced FKBP3. The PI was used to dye. (C) The protein of cyclin D1, cyclin B1, CDK4 and CDK1 was measured by western blot analysis. Data were expressed as mean ± SD (*n* = 3). ^#^ *p* < 0.05 compared to vector 1 or siNC. ^##^ *p* < 0.01 compared to vector 1 or siNC. CDK4, cyclin dependent kinase 4; DLBCL, diffuse large B‐cell lymphoma; FKBP3, FK506 binding protein 3; PI, propidium iodide; SD, standard deviation.

Flow cytometry was used to detect the influence of FKBP3 on the cell cycle. As shown in Figure 2A, the percentages of cells in G1 were markedly reduced after transfection with oeFKBP3 but significantly increased after siFKBP3 transfection (Figure 2B). Protein expression of cyclin D1, cyclin B1, CDK4 and CDK1 were higher in oeFKBP3 transfected cells, but lower in siFKBP3 cells (Figure 2C). These data demonstrated that FKBP3 silencing caused the arrest of G1.

### 3.3 FKBP3 stimulated the expression of stemness markers in DLBCL cells

**FIGURE 3:**
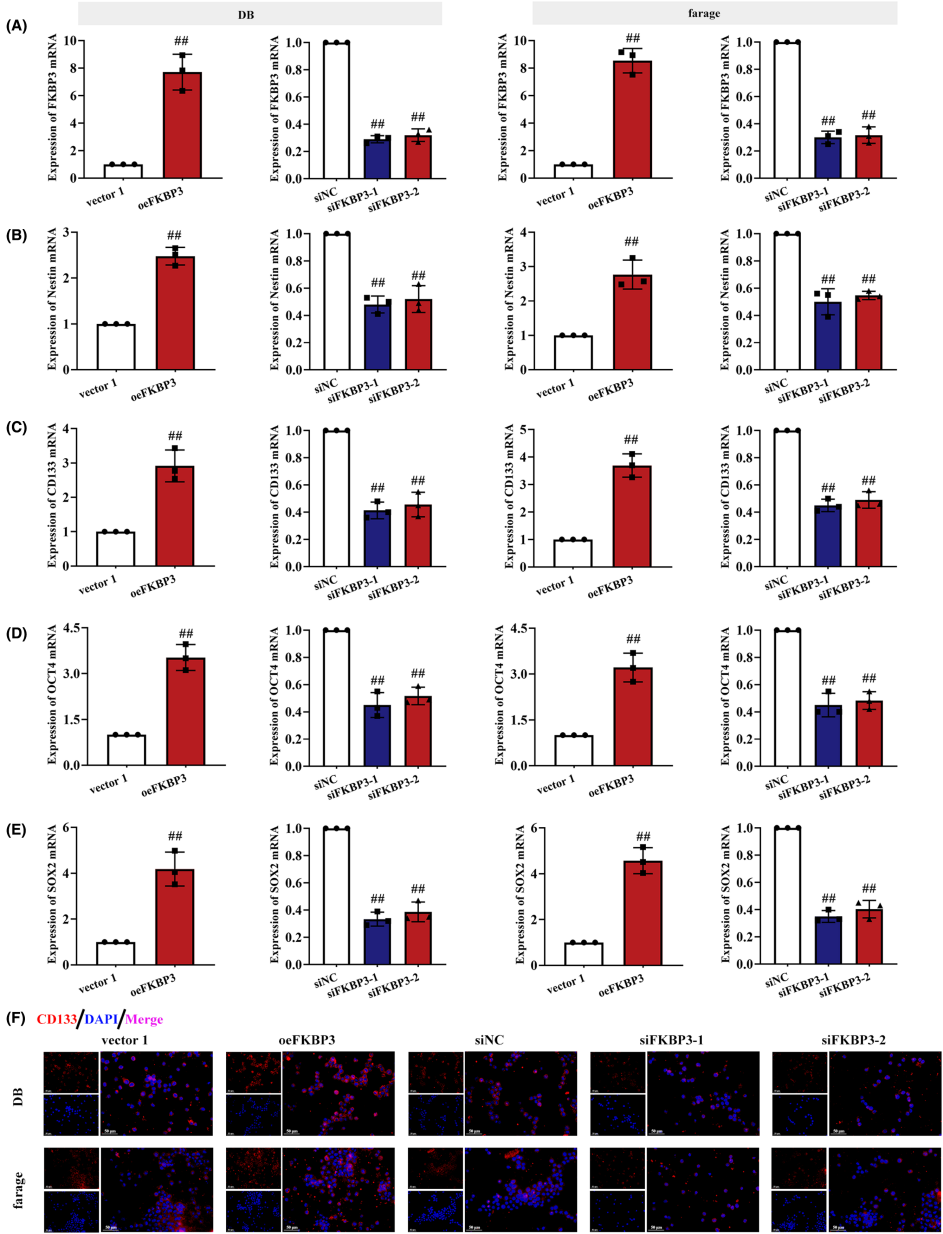
FKBP3 stimulated the expression of stemness markers in DLBCL cells. The mRNA of FKBP3 (A), Nestin (B), CD133 (C), OCT4 (D) and SOX2 (E) in DB cells and farage cells was analysed by real‐time PCR. (F) The CD133 (red) protein expression was measured by immunofluorescence. Nucleus was stained blue with DAPI in DB cells and farage cells. Bars, 50 μm, ×400. Data were expressed as mean ± SD (*n* = 3). ^##^ *p* < 0.01 compared to vector 1 or siNC. CD133, prominin 1; DAPI, 4′,6‐diamidino‐2‐phenylindole; DLBCL, diffuse large B‐cell lymphoma; FKBP3, FK506 binding protein 3; OCT4, POU class 5 homeobox 1; PCR, polymerase chain reaction; SD, standard deviation; SOX2, SRY‐Box transcription factor 2.

Stemness of tumour includes the ability to self‐renew and the potential to develop into tumours, and the regulatory processes of controlling stemness are active in cancer. Therefore, we also checked whether FKBP3 affected the stemness of DLBCL cells. The expression of FKBP3 mRNA in cells transfected with oeFKBP3 was high, while that in cells transfected with siFKBP3 was low (*p* < 0.01; Figure 3A). The expression of stemness markers (Nestin, CD133, OCT4 and SOX2) was increased in oeFKBP3 cells but reduced in siFKBP3 cells (*p* < 0.01; Figure 3B–F). These data suggested that FKBP3 enhanced the stemness of DLBCL cells.

### 3.4 FKBP3 boosted tumour growth of mice with DLBCL xenograft

**FIGURE 4:**
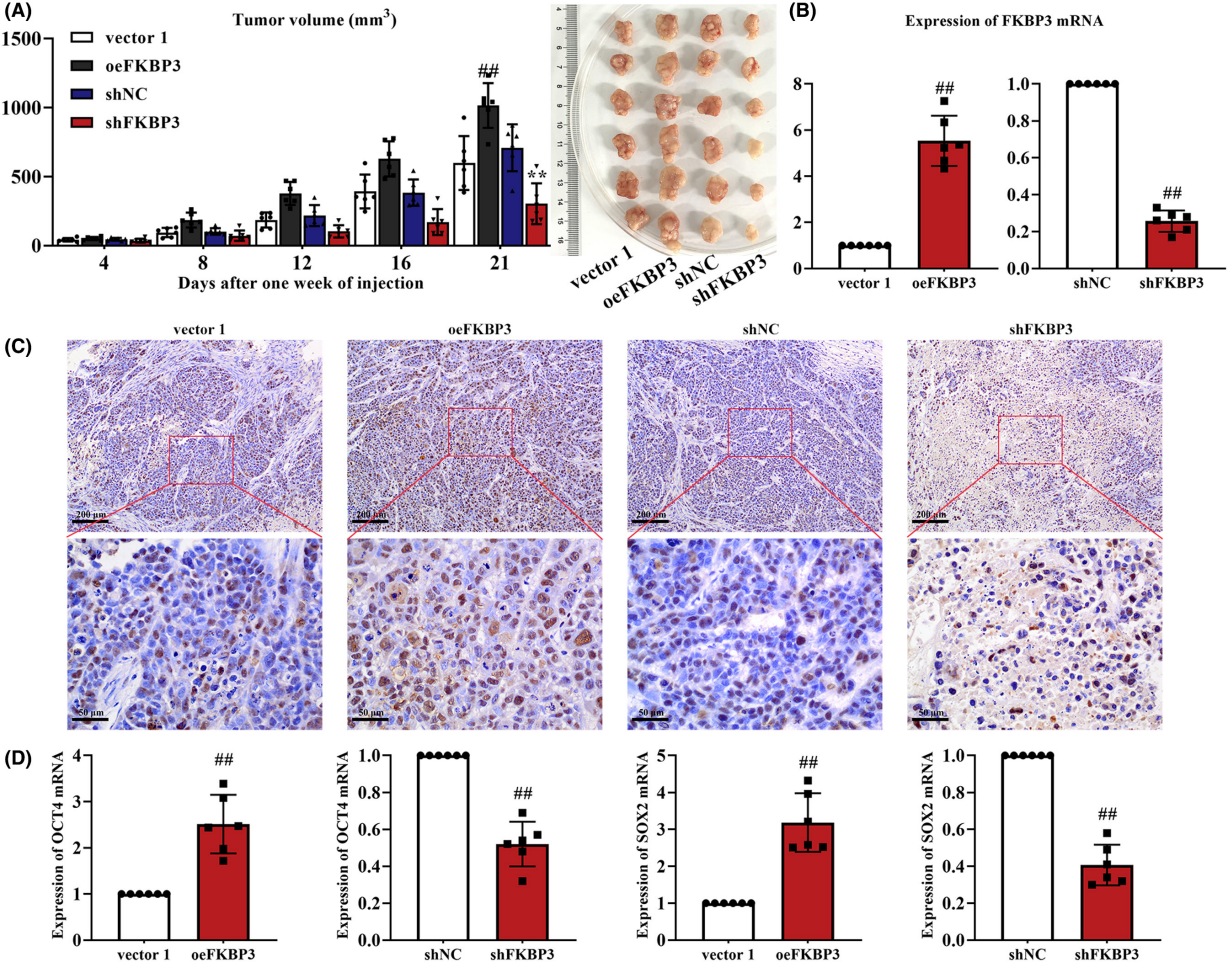
FKBP3 boosted tumour growth of mice with DLBCL xenograft. (A) Tumour volume on day 4, 8, 12, 16 and 21, and tumour images on day 21 in each group were shown. (B) Real‐time PCR was used to detect the mRNA expression of FKBP3 in tumour. (C) IHC was used to detect the PCNA expression in tumour. Lower panels showed the magnified images of regions framed by red rectangles on the upper panels. Bars, 200 or 50 μm, ×100 or ×400. (D) The mRNA expression of OCT4 and SOX2 was measured by real‐time PCR. Data were expressed as mean ± SD (*n* = 6). ^##^ *p* < 0.01 compared to vector 1 or shNC. **p < 0.01 compared to shNC. DLBCL, diffuse large B‐cell lymphoma; FKBP3, FK506 binding protein 3; IHC, immunohistochemistry; OCT4, POU class 5 homeobox 1; PCNA, proliferating cell nuclear antigen; PCR, polymerase chain reaction; SD, standard deviation; SOX2, SRY‐Box transcription factor 2.

We above confirmed that FKBP3 had a direct influence on the survival and function of DLBCL cells. To further identify the role of FKBP3 on DLBCL in vivo, we next used a nude mouse xenograft model. Tumour growth in mice implanted with oeFKBP3 cells was rapid and tumour volume was larger at day 21 than in mice implanted with vector 1 cells. However, knockdown of FKBP3 treatment significantly reduced tumour volume (*p* < 0.01; Figure 4A). Mice with xenograft of oeFKBP3 DLBCL cells showed high expression of FKBP3 mRNA and shFKBP3 mice displayed low mRNA expression of FKBP3 (*p* < 0.01; Figure 4B). PCNA levels were increased in mice with oeFKBP3 cells xenograft but decreased in shFKBP3 xenograft mice (Figure 4C). It showed that the proliferative activity was promoted by FKBP3. The mRNA expression of OCT4 and SOX2 was also increased in oeFKBP3 mice and decreased in shFKBP3 mice (*p* < 0.01; Figure 4D) suggesting that the stemness was enhanced by FKBP3. All these results revealed that FKBP3 facilitated the growth of tumour in vivo.

### 3.5 FKBP3 promoted the stability of PARK7

**FIGURE 5:**
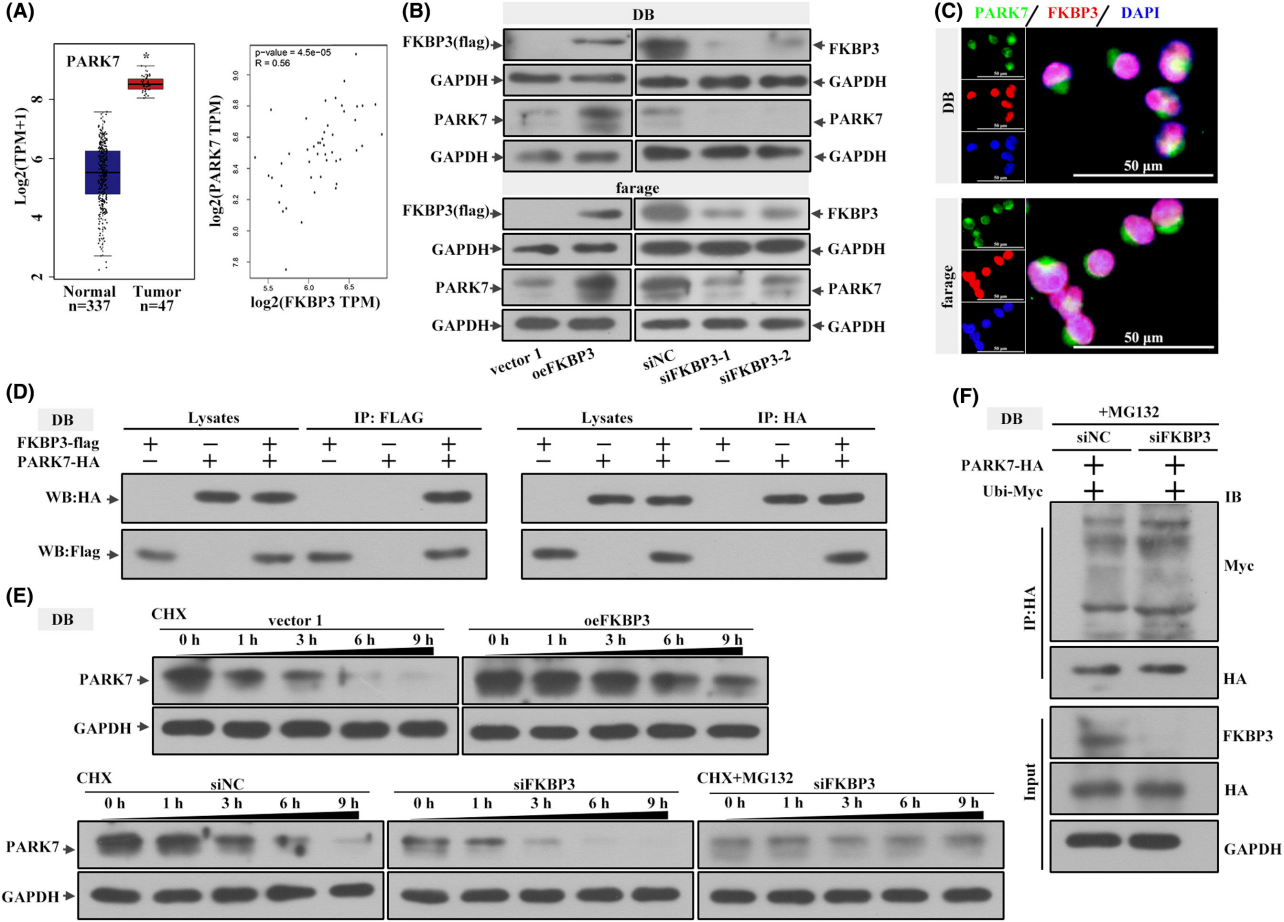
FKBP3 promoted the stability of PARK7. (A) PARK7 expression in DLBCL and paired normal sample in the GEPIA database. The correlation analysis of PARK7 and FKBP3. (B) Western blot analysis was used to detect the protein expression of FKBP3 and PARK7 in DB cells and farage cells. (C) The expression and localization of FKBP3 (red) and PARK7 (green) in DB cells and farage cells were determined by immunofluorescence assay. The nucleus was stained blue with DAPI. Bars, 50 μm, ×400. (D) CoIP was used to detect the interaction between FKBP3 and PARK7 in DB cells. (E) The protein expression of PARK7 in DB cells after treatment with CHX or CHX + MG132 for 0, 1, 3, 6 and 9 h was measured by western blot analysis. (F) The binding of PARK7 and Ubi was analysed by CoIP in DB cells. Data were expressed as mean ± SD (*n* = 3). *P < 0.05 compared to normal samples. CHX, cycloheximide; CoIP, co‐immunoprecipitation; DAPI, 4′,6‐diamidino‐2‐phenylindole; FKBP3, FK506 binding protein 3; PARK7, parkinsonism associated deglycase; SD, standard deviation; Ubi, ubiquitin.

Since FKBP3 and PARK7 were highly expressed and correlated in DLBCL, we hypothesized that PARK7 might be a downstream factor regulated by FKBP3. To study the correlation between FKBP3 and PARK7, we performed further experiments and found the following results. GEPIA data showed that PARK7 expression was also high in DLBCL. Interestingly, the expression of PARK7 was positively correlated with FKBP3 expression (Figure 5A). The expression of FKBP3 and PARK7 was high in DLBCL cells transfected with oeFKBP3 and low in DLBCL cells transfected with siFKBP3 (Figure 5B). We observed nuclear co‐localization of FKBP3 and PARK7 in Figure 5C. In cells transfected with oeFKBP3 and oePARK7, PARK7 (HA) was detected in material immunoprecipitated with FKBP3 (FLAG). Reciprocal HA immunoprecipitation followed by FLAG immunoblotting confirmed the association of FKBP3 and PARK7 (Figure 5D). It indicated that FKBP3 interacted with PARK7 in DB cells. We chose the more effective FKBP3 siRNA for subsequent experiments. Protein expression of PARK7 was gradually reduced with the extension of CHX treatment time. But MG132 effectively inhibited the degradation of PARK7 (Figure 5E). According to the results in Figure 5F, the ubiquitination modification of PARK7 was increased in siFKBP3 silenced cells. These results demonstrated that FKBP3 inhibited the ubiquitination modification of PARK7 and reduced its degradation.

### 3.6 PARK7 activated Wnt/β‐catenin signalling pathway and enhanced the stemness of DLBCL cells

**FIGURE 6:**
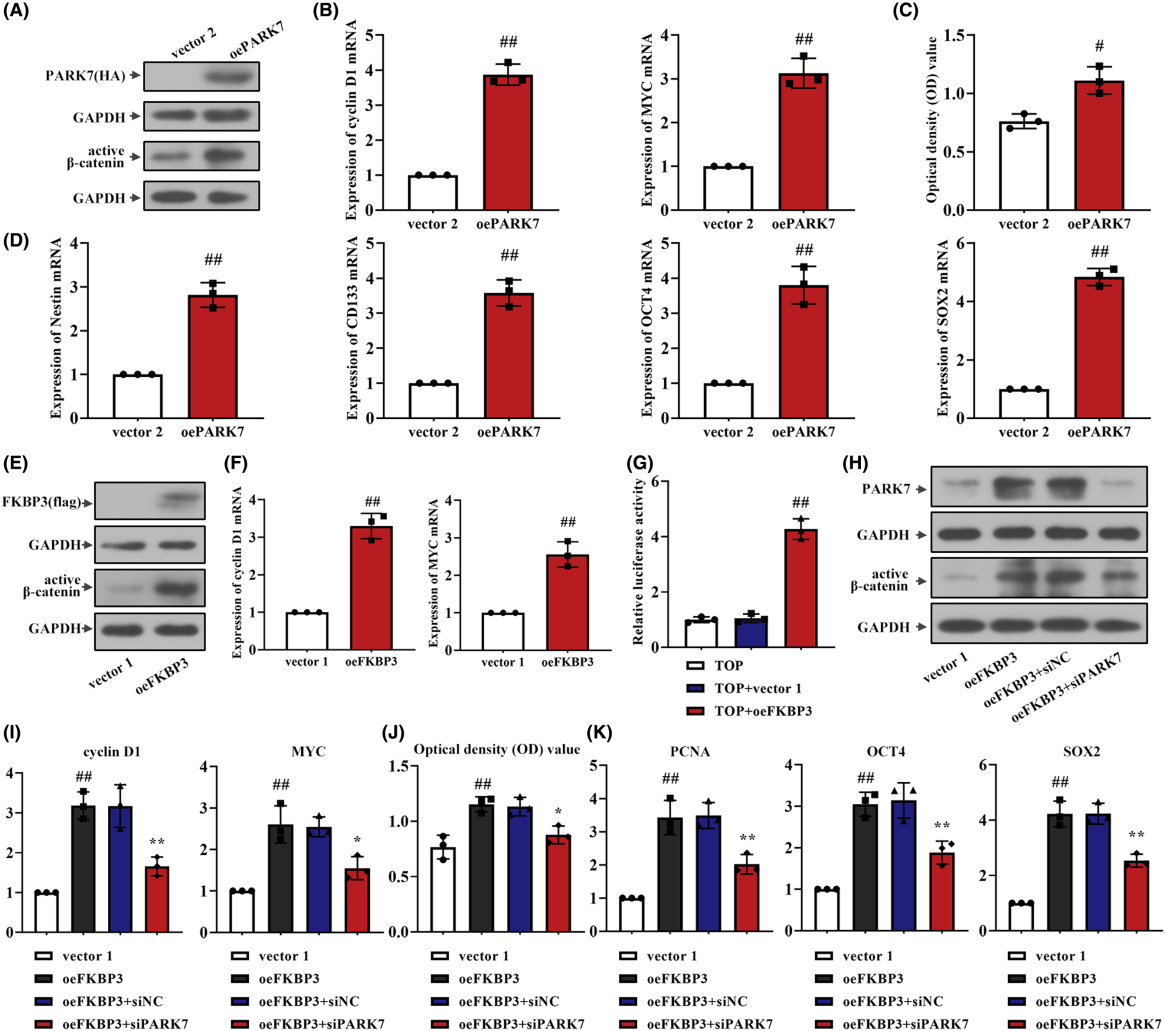
FKBP3 activated Wnt/β‐catenin signalling pathway and aggravated the malignant phenotype of DLBCL cells through increasing PARK7 expression. (A) The protein expression of PARK7 and active β‐catenin was measured by western blot analysis. (B) Real‐time PCR was used to detect the mRNA expression of cyclin D1 and MYC in DB cells. (C) Cell viability of DB cells was analysed by CCK‐8. The optical density value meant the cell viability. (D) The mRNA expression of Nestin, CD133, OCT4 and SOX2 was measured by Real‐time PCR. (E) Western blot analysis was used to detect the protein expression of FKBP3, active β‐catenin in DB cells. (F) The mRNA expression of cyclin D1 and MYC in DB cells was analysed by real‐time PCR. (G) The transcriptional activity of β‐catenin was detected by luciferase reporter assay. (H) The protein expression of PARK7 and active β‐catenin was measured by western blot analysis. (I) Real‐time PCR was used to detect the mRNA expression of cyclin D1 and MYC in DB cells. (J) Cell viability of DB cells was analysed by CCK‐8. The optical density value meant the cell viability. (K) The mRNA expression of PCNA, OCT4 and SOX2 was measured by Real‐time PCR. Data were expressed as mean ± SD (*n* = 3). ^#^ *p* < 0.05 compared to vector 2. *p < 0.05 compared to normal or oeFKBP3 + siNC. ^##^ *p* < 0.01 compared to vector 1, vector 2 or TOP +vector 1. **p < 0.01 compared to oeFKBP3 + siNC. CCK‐8, cell counting kit‐8; CD133, prominin 1; DLBCL, diffuse large B‐cell lymphoma; FKBP3, FK506 binding protein 3; MYC, MYC Proto‐Oncogene, BHLH Transcription Factor; OCT4, POU class 5 homeobox 1; PARK7, parkinsonism associated deglycase; PCNA, proliferating cell nuclear antigen; PCR, polymerase chain reaction; SD, standard deviation; SOX2, SRY‐Box transcription factor 2.

More and more evidence suggests that Wnt/β‐catenin contributes to cancer progression, and previous study also confirmed that PARK7 activated the Wnt pathway, thereby promoting cancer development. In order to determine the effect of PARK7 on the Wnt/β‐catenin signalling pathway in DLBCL, we measured the expression of Wnt‐related molecules. As shown in Figure 6A, protein expression of PARK7 and active β‐catenin was high in cells transfected with oePARK7. The expression of cyclin D1 and MYC mRNA was elevated in oePARK7 cells (*p* < 0.01; Figure 6B). These results demonstrated that the Wnt/β‐catenin signalling pathway was activated by PARK7. The activity of cells transfected with oePARK7 was significantly increased (*p* < 0.05; Figure 6C). Increased expression of Nestin, CD133, OCT4 and SOX2 mRNA was also shown in cells transfected with oePARK7 (*p* < 0.01; Figure 6D). These data suggested that the malignant phenotype of DLBCL cells was exacerbated by PARK7.

### 3.7 FKBP3 activated Wnt/β‐catenin signalling pathway and aggravated the malignant phenotype of DLBCL cells through increasing PARK7 expression

Based on the above results, we concluded that PARK7 activated the Wnt/β‐catenin signalling pathway and subsequently increased the malignant phenotype. However, the effect of FKBP3 on Wnt/β‐catenin signalling pathway was unknow. To clarify whether FKBP3 affected DLBCL malignant phenotype through the Wnt/β‐catenin signalling pathway, we examined changes in Wnt‐related molecules expression and DLBCL malignant phenotype. Cells with oeFKBP3 transfection showed elevatory FKBP3 protein expression, accompanied with increased active β‐catenin (Figure 6E). Also, the mRNA expression of cyclin D1 and MYC was higher in oeFKBP3 cells (*p* < 0.01; Figure 6F). FKBP3 induced an approximately four‐fold higher transcription of β‐catenin than vector 1 (*p* < 0.01; Figure 6G). It indicated that FKBP3 activated the Wnt/β‐catenin signalling pathway. Protein expression of PARK7 and active β‐catenin was enhanced in cells transfected with oeFKBP3, while the protein expression was decreased in oeFKBP3 and siPARK7‐cotransfected cells (Figure 6H). Consistently, high mRNA expression of cyclin D1 and MYC in oeFKBP3 cells was reduced by co‐transfection of oeFKBP3 and siPARK7 (Figure 6I). The cell viability was increased in oeFKBP3‐treated cells but decreased after PARK7 silencing (Figure 6J). Moreover, downregulation of PARK7 reduced FKBP3‐enhanced PCNA, OCT4 and SOX2 mRNA expression (*p* < 0.01; Figure 6K). These results demonstrated that PARK7 mediated the function of FKBP3 on Wnt/β‐catenin signalling pathway, thereby intensifying the malignant behaviour of DB cells.

### 3.8 FKBP3 transcription was suppressed by FOXO3

**FIGURE 7:**
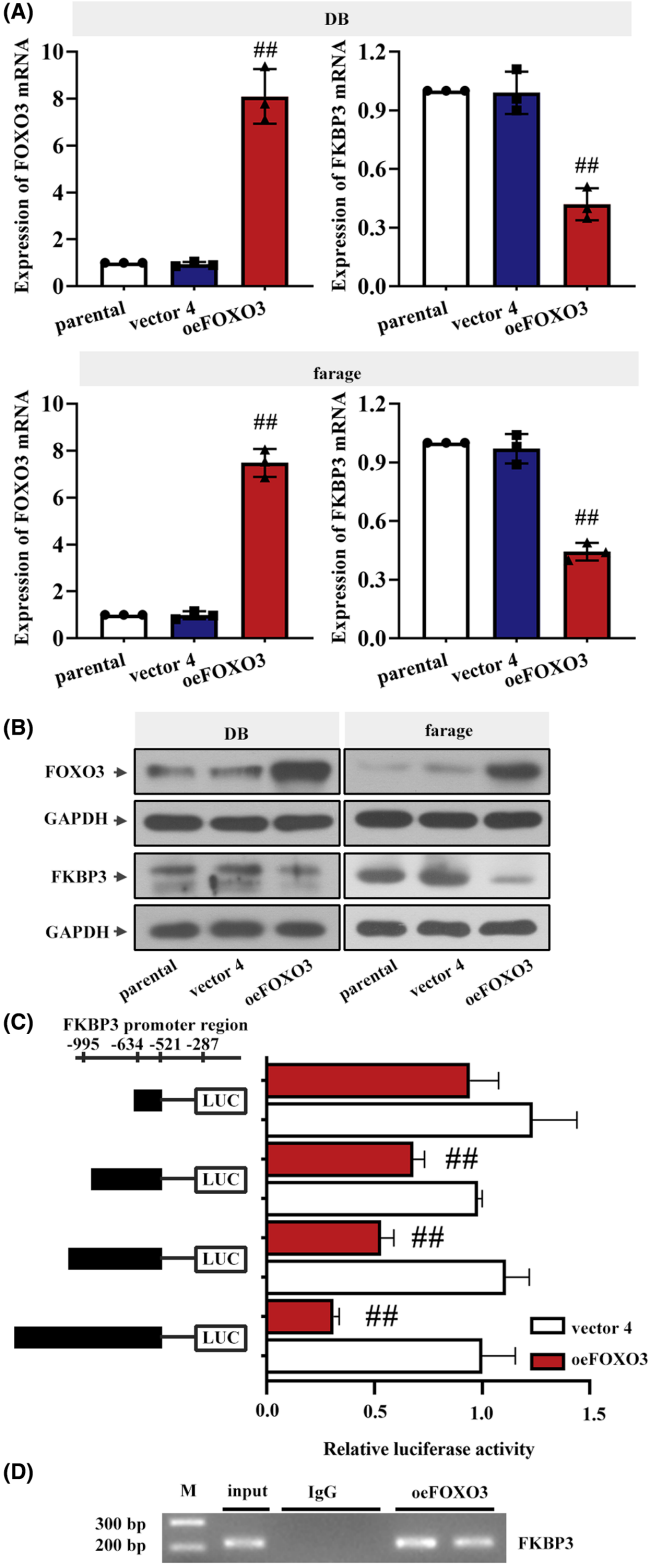
FKBP3 transcription was suppressed by FOXO3. (A) Real‐time PCR and (B) western blot analyses were used to detect the mRNA and protein expression of FOXO3 and FKBP3 in DLBCL cells. (C) FKBP3 transcription in oeFOXO3‐transfected DB cells was measured by luciferase reporter assay. The region of FKBP3 promoter was cloned into the pGL3 reporter vector. (D) ChIP was performed to detect the binding of FOXO3 to FKBP3 promoter in DB cells. Data were expressed as mean ± SD (*n* = 3). ^##^ *p* < 0.01 compared to vector 4 or vector 4 + pGL3 promoter (−995/+19, −634/+19, −521/+19). ChIP, chromatin immunoprecipitation; DLBCL, diffuse large B‐cell lymphoma; FKBP3, FK506 binding protein 3; FOXO3, Forkhead Box O3; PCR, polymerase chain reaction; SD, standard deviation.

Because FOXO3 was reduced in DLBCL and its expression appeared to be negatively correlated with FKBP3 expression, it is worth investigating how FOXO3 affects FKBP3 and alters DLBCL. To find the relationship between FKBP3 and FOXO3, we performed real‐time PCR, western blot, luciferase reporter and ChIP assays. As shown in Figure 7A,B, the mRNA and protein expression of FOXO3 was increased in oeFOXO3‐transfected cells. Interestingly, overexpression of FOXO3 decreased the mRNA and protein levels of FKBP3 (Figure 7A,B). According to the results of Jaspar analysis, there may be binding sties of FOXO3 in the promoter of FKBP3. Thus a luciferase reporter assay was conducted to evidence the regulatory effect of FOXO3 on the transcription of FKBP3. When the promoter region was inserted into the pGL3 vector, the transcription was inhibited by FOXO3. While this inhibitory effect was gradually attenuated along with the truncation of the promoter region (Figure 7C). By ChIP assay, we found that FOXO3 bound to the promoter region of FKBP3 (Figure 7D). These results demonstrated that FOXO3 suppressed the transcription of FKBP3.

## 4 DISCUSSION

DLBCL is a lymphoid malignancy consisting of cells with prominent nucleoli, vesicular nuclei, basophilic cytoplasm and high proliferation rate. The overall survival of patients with DLBCL is regrettable.^17^ The search for potential therapeutic targets is meaningful for the effective treatment of DLBCL. Our results elucidated the high expression and oncogenic role of FKBP3 in DLBCL. Some members of FKBPs participate in carcinogenesis.^5^ Previous studies showed increased expression of FKBP3 in lymphoma‐related cells.^8^, ^20^ Our results demonstrated that FKBP3 was conducive to the malignant phenotype and stemness of DLBCL, and promoted the tumour growth in a xenograft mouse model. However, knockdown of FKBP3 exhibited the opposite results. These results were similar to many findings. FKBP3 facilitated the proliferation of NSCLC cells.^7^ Knockdown of FKBP3 restrained proliferation and invasion of breast cancer cells, and induced apoptosis of breast cancer.^21^ High level of FKBP3 is related to poor survival in lung adenocarcinoma (LUAD) patients.^22^ FKBP3 is located in the nucleus, binds to nucleic acids and combines with chromatin modifying enzymes.^23^ Dilworth et al. proposed that FKBP3 was associated with the mitotic spindle. We found that FKBP3 silencing caused cell cycle arrest in DLBCL cells as evidenced by the reduced S‐phase content and increased G1 population, which was similar to previous study.^24^ These findings indicated that the proliferation of DLBCL cells was inhibited by knockdown of FKBP3.

Next, we check whether FKBP3 influenced other functions of DLBCL cells, including stemness. Stemness is a functional definition that allows cancer cells to reach distant organs and cause tumour metastasis.^25^, ^26^ FKBP3 promoted the stemness of DLBCL cells, whereas FKBP3 silencing attenuated cell stemness. It indicated that FKBP3 may contribute to tumour metastasis. Expression of core stemness‐related transcription factors (CD133, SOX2, OCT4) was increased in DLBCL cells.^27^ PARK7 was co‐expressed with Nestin during neural stem cells (NSCs) proliferation.^28^ We found that the stemness of DLBCL cells was enhanced after PARK7 overexpression vector transfection. Kim et al. suggested that PARK7 knockdown suppressed the invasion of GSCs and expression of GSC signatures (Nestin, SOX2 and OCT4), thereby reducing the stemness of GSCs.^12^ When human astrocytes were reprogramed into neural stem cell‐like cells expressing Nestin and SOX2, these cells would differentiate and form tumorospheroids.^29^ Our results indicated that FKBP3 and PARK7 increased the stemness of DLBCL cells accordingly promoting tumour growth, and they were positively correlated.

Previous studies suggested that FKBP3 plays a specific role in the regulation of protein ubiquitination, which in turn affects cancer progression.^7^, ^30^ We speculated that FKBP3 might regulate the ubiquitinylation modulation of PARK7 and performed further validation. FKBP3 delayed the degradation time of PARK7 and might influence the expression of PARK7. Degradation of proteins after ubiquitination is critical for many physiological processes, including oncogenesis and metabolism.^31^ In this process, ubiquitin (Ubi) is activated by Ubi activating enzyme (E1) and then transferred to Ubi conjugating enzyme (E2). Ubi ligating enzyme (E3) finally binds Ubi‐loaded E2 to substrate protein, thereby modifying the protein.^32^ Our results suggested that FKBP3 was combined with PARK7 and regulated the ubiquitination and degradation of PARK7, accordingly influencing the malignant phenotype of DLBCL.

It was worth exploring how the transcription of FKBP3 was regulated. In MYC‐driven lymphomagenesis, FOXO3 had a remarkable tumour‐suppressor function.^33^ Bisserier et al. proposed that FOXO3 was required for antitumor effects.^34^ The expression of FKBP3 was increased in B‐cell lymphomas.^8^ FOXO3 has cancer suppressive properties, while FKBP3 is carcinogenic. After analysis with Jaspar, there were potential binding sites for FOXO3 in the promoter of FKBP3, hence we wondered whether FOXO3 modulated the transcription of FKBP3. Consistent with these findings, our results indicated that the transcription of FKBP3 was suppressed by FOXO3, thereby attenuating DLBCL.

Activated Wnt/β‐catenin signalling pathway causes the transcription of genes implicated in cell proliferation, protein synthesis and tumour growth. The pathway is crucial in the development of numerous cancers and is activated in DLBCL.^35^, ^36^, ^37^ Aberrant Wnt/β‐catenin pathway results in β‐catenin accumulation, which in turn led to nuclear translocation of β‐catenin and activation of the expression of target genes (cyclin D1 and MYC).^38^ Our results demonstrated that FKBP3 activated the Wnt/β‐catenin signalling pathway, consequently increasing the expression of cyclin D1 and MYC. It revealed that FKBP3 enhanced the malignant phenotype of DLBCL through the Wnt/β‐catenin signalling pathway. Previous studies suggested that after activation of the Wnt/β‐catenin signalling pathway by PARK7, cancer progression was promoted.^14^, ^15^ Our data revealed that FKBP3 activated the Wnt/β‐catenin pathway through regulating the ubiquitination and degradation of PARK7 (Figure S2).

## 5 CONCLUSION

FKBP3 activated the Wnt/β‐catenin signalling pathway and aggravated the malignant phenotype of DLBCL cells. FKBP3 combined with PARK7 and suppressed its ubiquitination, thereby inhibiting its degradation. FOXO3 inhibited FKBP3 transcription and then suppressed the expression of FKBP3.

## AUTHOR CONTRIBUTIONS

**Xiaojing Xing:** Conceptualization (lead); data curation (lead); funding acquisition (lead); methodology (lead); writing – original draft (lead). **Meichen Liu:** Data curation (equal). **Xuguang Wang:** Data curation (equal). **Qianxue Guo:** Data curation (equal). **Hongyue Wang:** Data curation (equal). **Wenxue Wang:** Conceptualization (equal).

## FUNDING INFORMATION

This work was supported by the National Natural Science Foundation of China (U1908215), the General Program of the National Natural Science Foundation of China (62273330), the Fundamental Research Funds for the Central Universities, the Provincial and Ministerial Science Foundation (Millions of Talents Project of Liaoning Province in 2019).

## CONFLICT OF INTEREST STATEMENT

The authors declare that they have no competing interests.

## Supporting information


Figure S1

Figure S2

## Data Availability

Data available on request from the authors.
