# Supplementary material for: FKBP3 aggravates the malignant phenotype of diffuse large B‐cell lymphoma by PARK7‐mediated activation of Wnt/β‐catenin signalling
Source: J Cell Mol Med. 2023 Nov 21;28(1):e18041. doi: 10.1111/jcmm.18041 (PMC10805489; doi:10.1111/jcmm.18041)
Supplement: Supplementary file 1 — Figure S1 [file JCMM-28-e18041-s001.zip › jcmm18041-sup-0002.FigureS1.docx]

**Figure S1.** Bioinformatics analysis of DLBCL dataset in GEPIA. (A) Heat map of differential gene expression with tumour and normal. GO (B) and KEGG (C) analysis of the differential genes between tumour and normal. (D) The location of the differential genes on chromosome was showed in the Circos plot. FKBP3 expression in DLBCL and paired normal sample in the GEPIA database. GEPIA, Gene Expression Profiling Interactive Analysis; FKBP3, FK506 binding protein 3; BP, biological process; CC, cellular component; MF, molecular function.
