# Supplementary figures and images for: FKBP3 aggravates the malignant phenotype of diffuse large B‐cell lymphoma by PARK7‐mediated activation of Wnt/β‐catenin signalling
Source: J Cell Mol Med. 2023 Nov 21;28(1):e18041. doi: 10.1111/jcmm.18041 (PMC10805489; doi:10.1111/jcmm.18041)

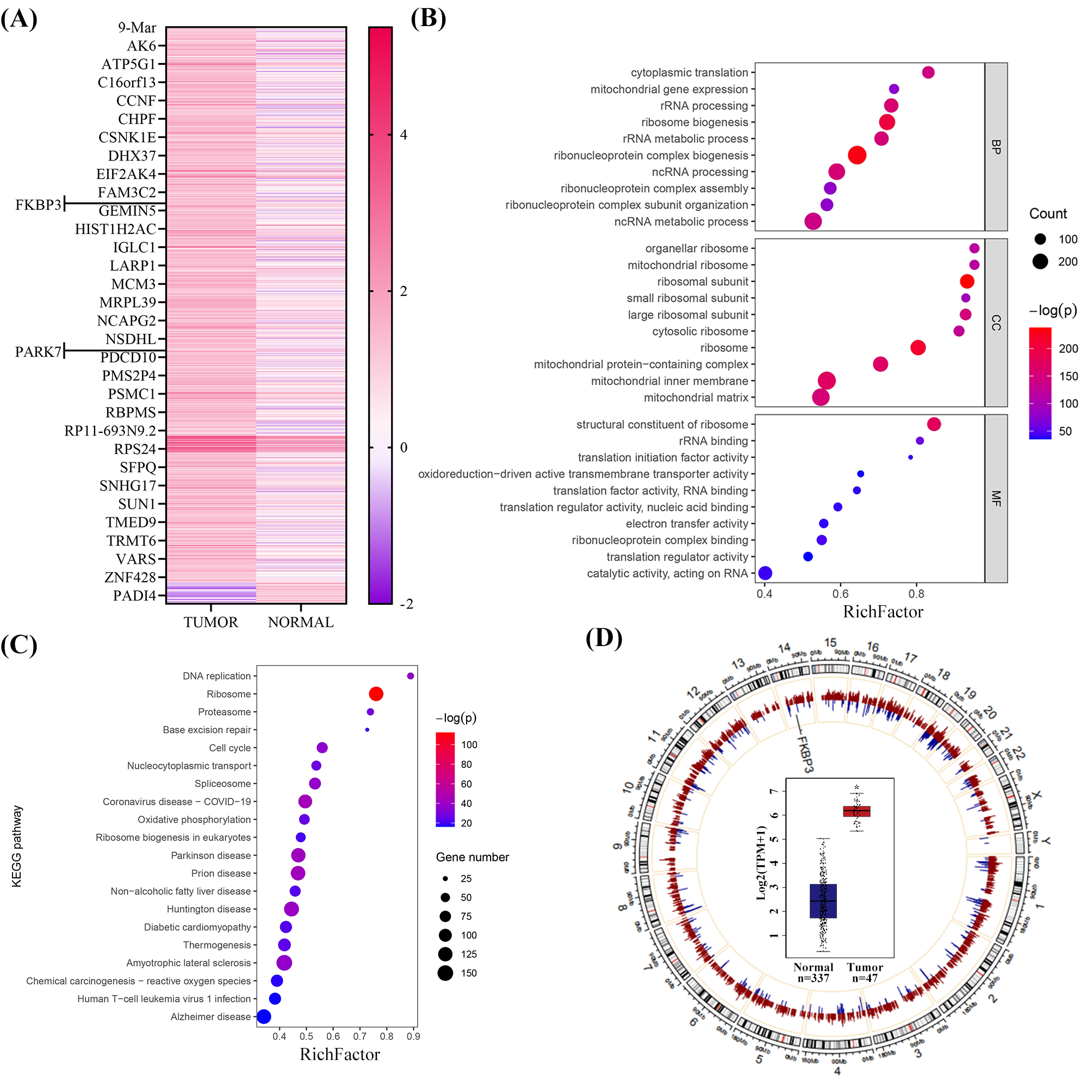

Supplement: Supplementary file 1 — Figure S1 [file JCMM-28-e18041-s001.zip › jcmm18041-sup-0001-FigureS1.tif]

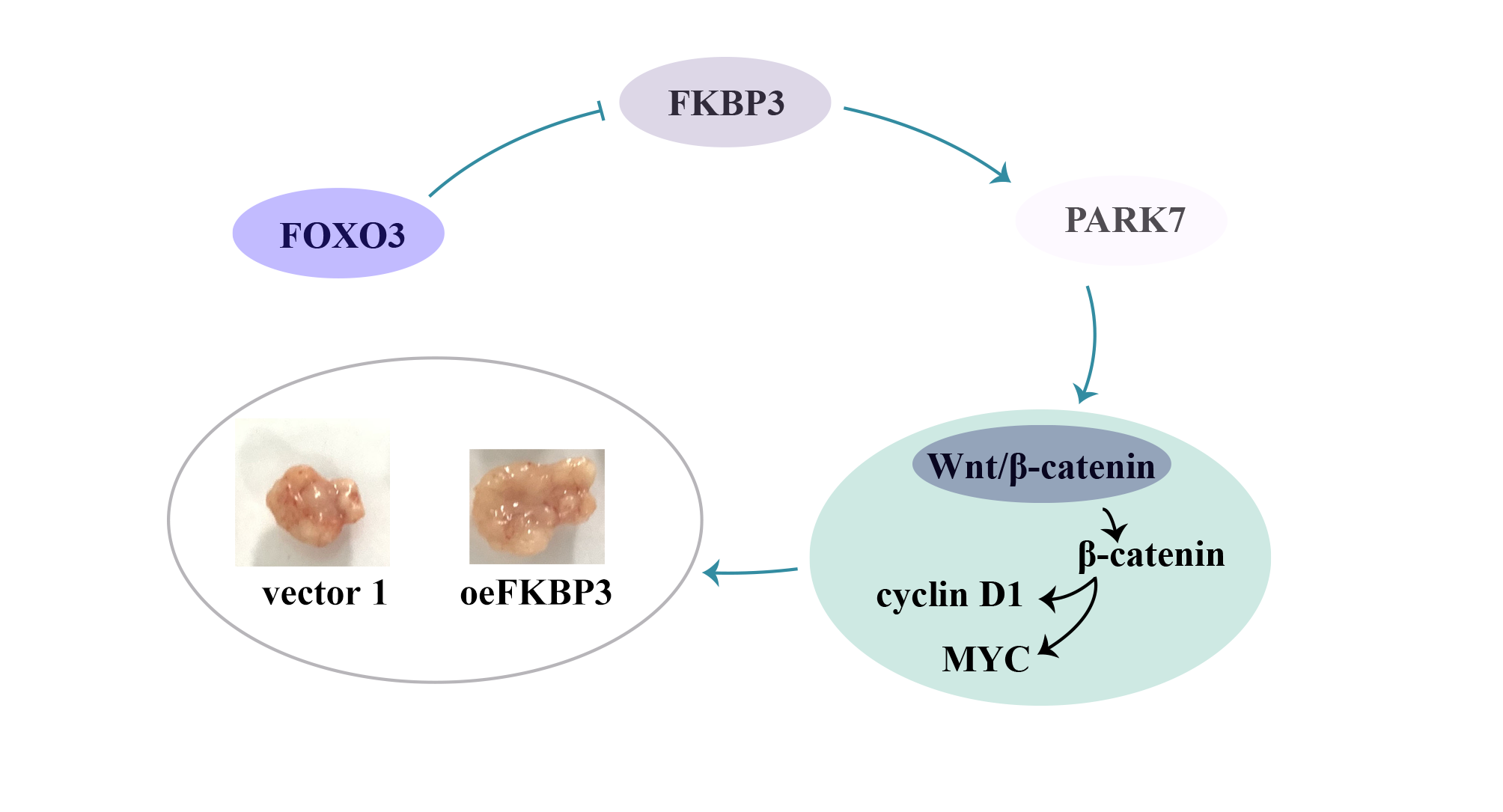

Supplement: Supplementary file 2 — Figure S2 [file JCMM-28-e18041-s002.zip › jcmm18041-sup-0002-FigureS2.tif]
